# Supplementary material for: MncR: Late Integration Machine Learning Model for Classification of ncRNA Classes Using Sequence and Structural Encoding
Source: Int J Mol Sci. 2023 May 17;24(10):8884. doi: 10.3390/ijms24108884 (PMC10218863; doi:10.3390/ijms24108884)
Supplement: Supplementary file 1 [file ijms-24-08884-s001.zip › Supplementaries.pdf]

See SUPPL_TABLE1.ods

Supplementary Table S1: Sequences in the RNAcentral test set falsely classified by any of the four models. Each column is one sequence that has been misclassified by either the StrEnc, *MncR*, GrEnc or SeqEnc model. For each sequence RNAcentral ID, RNA class, subclassification and length are provided. For sequences with no known or labeled subtype, “base” is given as the subtype. The table is sorted by firstly rna_type and then by amount of models that predicted correctly, meaning for each RNA type the first rows are the sequences falsely classified by all four models. Additionally, sequences classified by all models are also sorted according to whether all models agree on the classification or not. Color key is as following: Dark red: All models predict falsely and agree; Light red: All models predict falsely, but not all models agree on the prediction; Green: The corresponding model correctly predicts this sequence

See SUPPL_TABLE2.ods

Supplementary Table S2: Sequences in the Rfam benchmark test set falsely classified by either ncRDense or our *MncR* model. Each column is one sequence from the Rfam benchmark set that has been misclassified by either the *MncR* model or ncRDense. For each sequence Rfam ID, RNA class, label in the Rfam benchmark set, length, prediction by the *MncR* model and prediction by ncRDense are given. For the prediction of ncRDense, the predicted subtypes are renamed to their specific RNA class to match the labels of the *MncR* model.

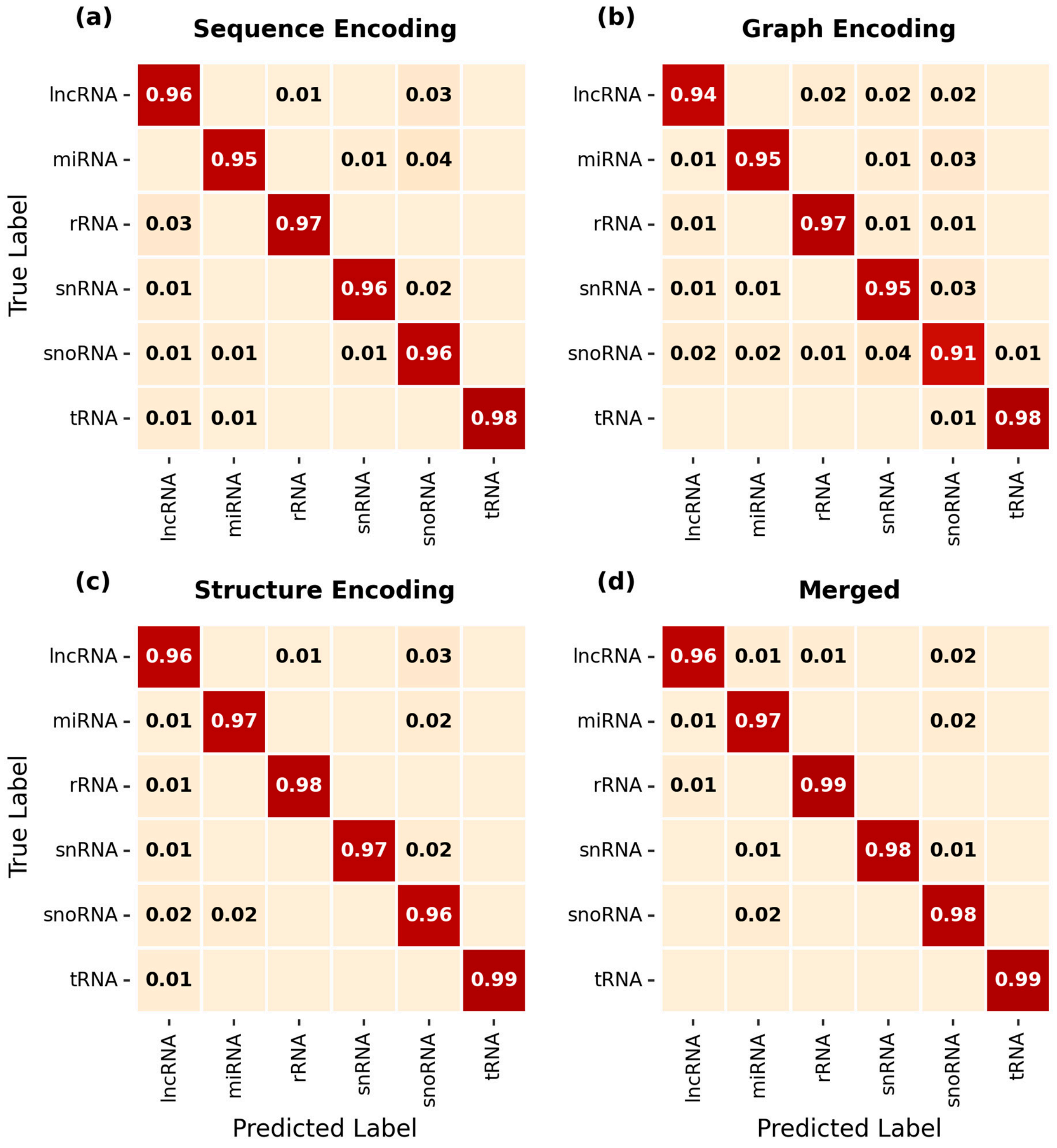

Supplementary Figure S1: Prediction differences for the four different models. Confusion matrices (normalized to each row) are shown with true labels as rows and predicted labels as columns. The fraction of predictions is indicated by the number in each square as well as the color gradient (low value: beige; high value: dark red). Values below 0.005 are omitted from the matrices. Values on the main diagonal in each matrix are equivalent to the recall for this class. A: SeqEnc. B: GrEnc. C: StrEnc. D: MncR.

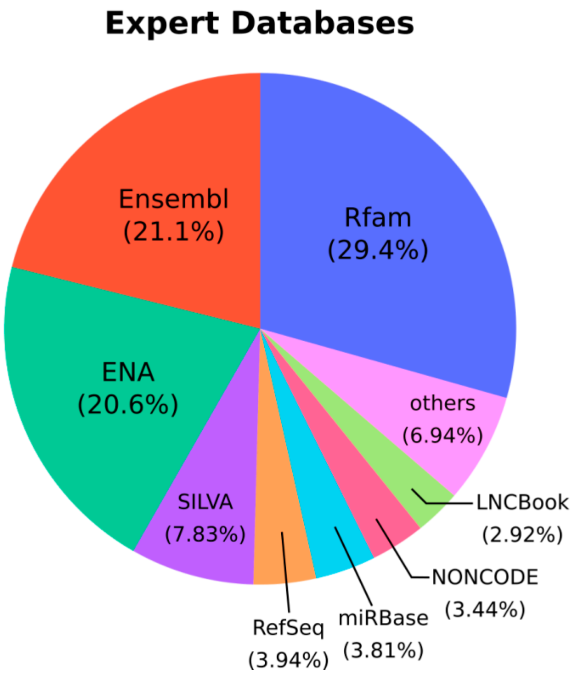

Supplementary Figure S2: Different Expert Databases represented within our RNAcentral data set. Pie chart of the percentages each Expert Database make up as the source of the RNAcentral set. Source databases were retrieved using the FTP archive from RNAcentral. For sequence IDs that map to multiple source databases, one was chosen at random. Total number of databases represented in our data set is 37.

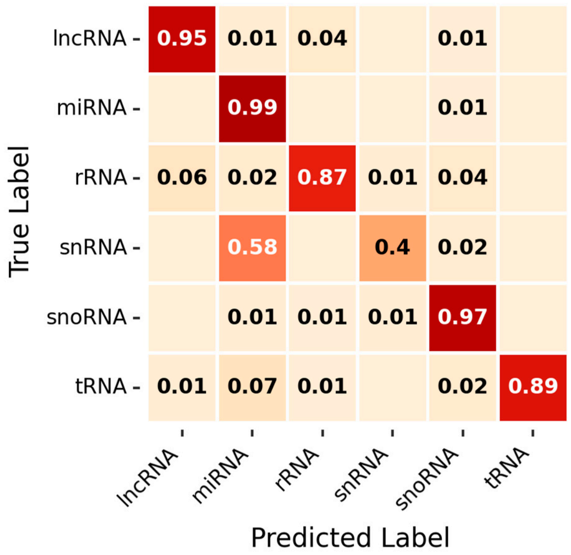

Supplementary Figure S3: Confusion matrix for the *MncR* prediction of the plant test set from NcodR. Confusion matrices (normalized to each row) are shown with the true label as rows and predicted labels as columns. The *MncR* predictions stem from the test set derived from sub-sampling the set used by NcodR to 166 sequences for each ncRNA class (996 total samples).
