# Supplementary figures and images for: MncR: Late Integration Machine Learning Model for Classification of ncRNA Classes Using Sequence and Structural Encoding
Source: Int J Mol Sci. 2023 May 17;24(10):8884. doi: 10.3390/ijms24108884 (PMC10218863; doi:10.3390/ijms24108884)

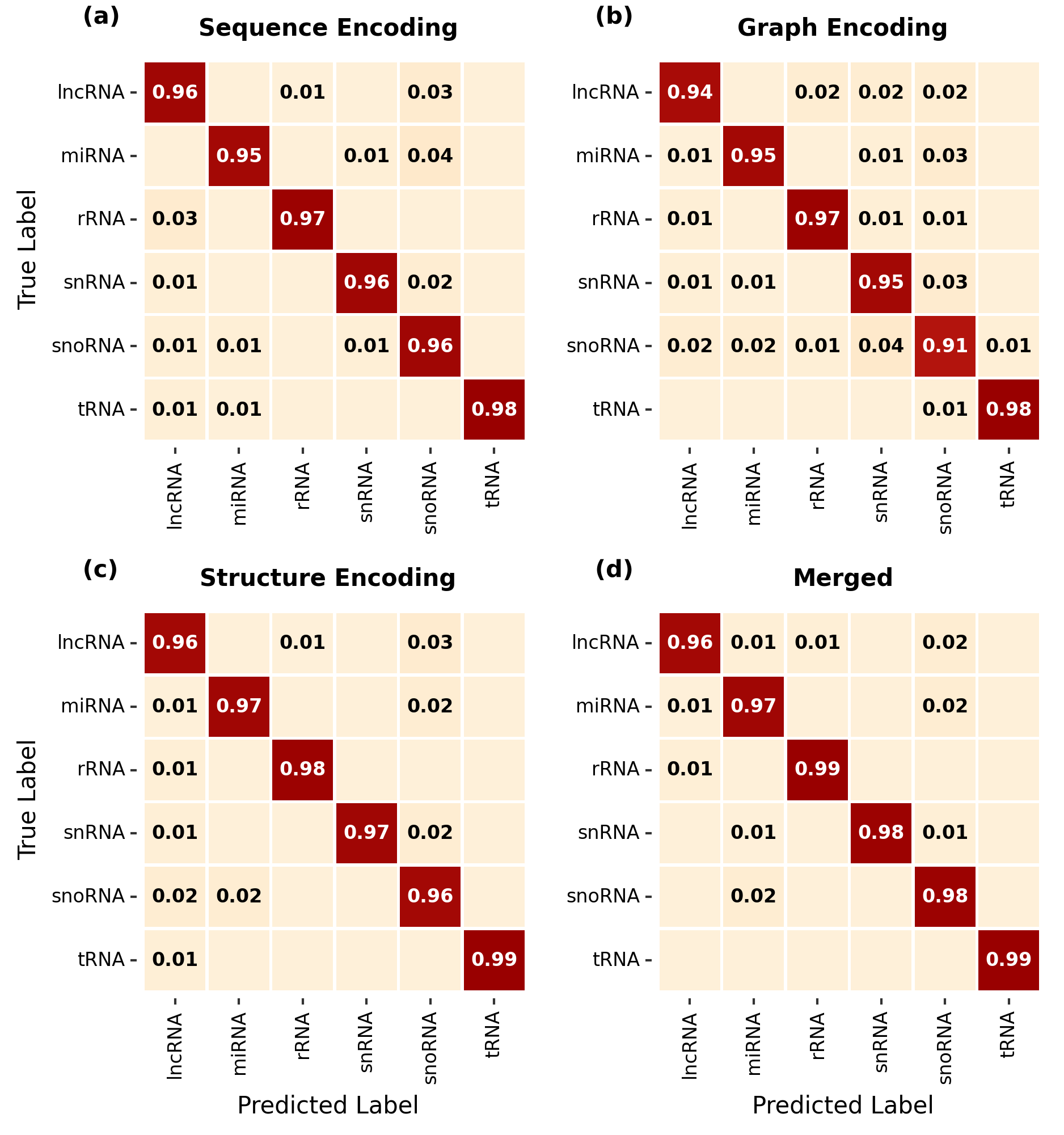

Supplement: Supplementary file 1 [file ijms-24-08884-s001.zip › SUPPL_FIG1.tif]

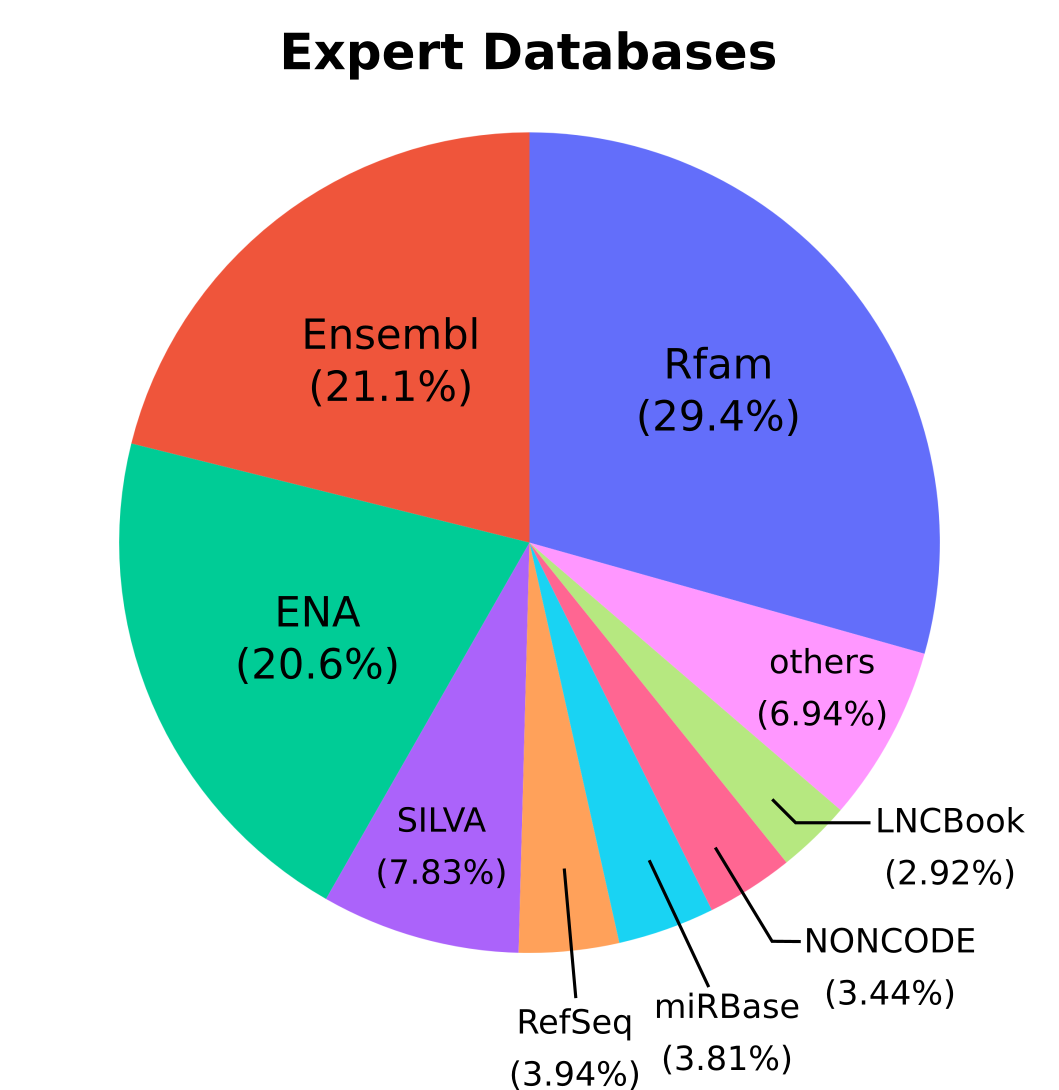

Supplement: Supplementary file 1 [file ijms-24-08884-s001.zip › SUPPL_FIG2.tif]

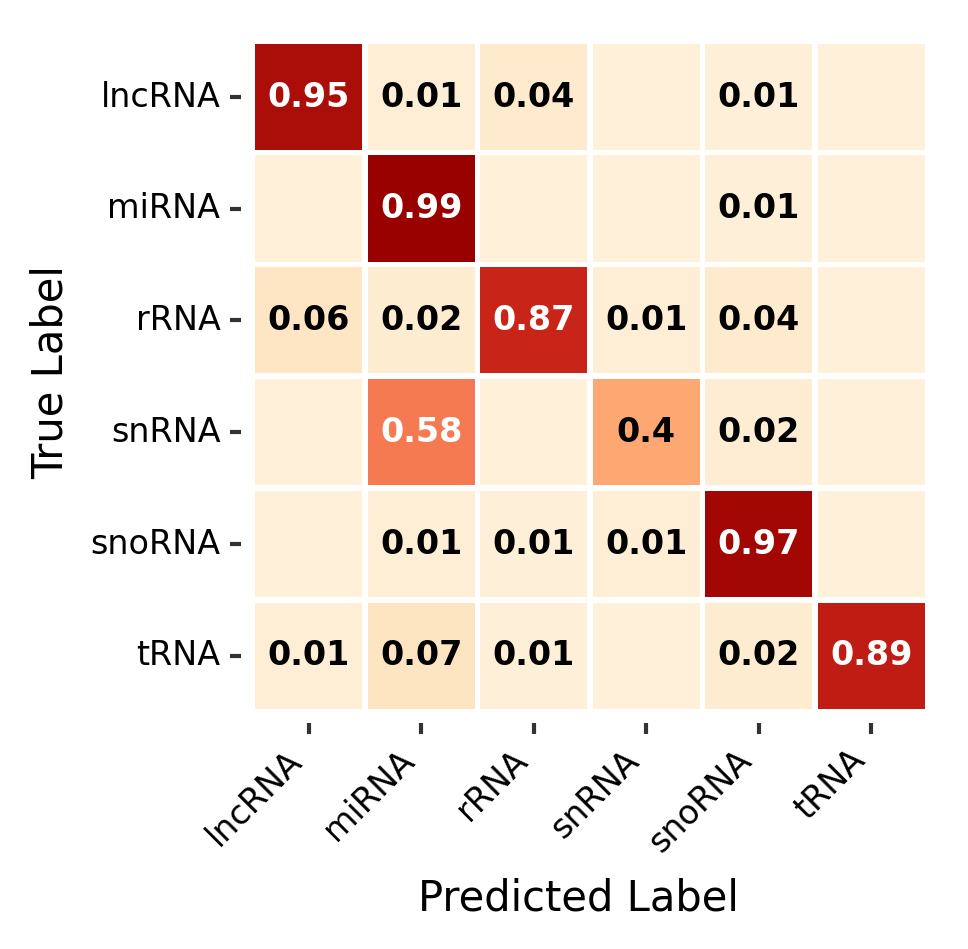

Supplement: Supplementary file 1 [file ijms-24-08884-s001.zip › SUPPL_FIG3.tiff]
